# Helping intentions of undergraduates towards their depressed peers: a cross-sectional study in Sri Lanka

**DOI:** 10.1186/s12888-017-1192-7

**Published:** 2017-01-23

**Authors:** Santushi D. Amarasuriya, Nicola J. Reavley, Alyssia Rossetto, Anthony F. Jorm

**Affiliations:** 1 Behavioural Sciences Stream, Faculty of Medicine, University of Colombo, PO Box 271, 25, Kynsey Road, Colombo 8, Sri Lanka; 2 Centre for Mental Health, Melbourne School of Population and Global Health, University of Melbourne, 207 Bouverie Street, Parkville, Victoria 3010 Australia

**Keywords:** Helping behaviours, Helping intentions, Mental health first aid, Mental health literacy, Depression, Undergraduate, University student, Sri Lanka

## Abstract

**Background:**

Despite showing high rates of depression, university students prefer to seek assistance for their depression from informal sources, such as their friends, rather than seeking professional assistance. Therefore, the helping behaviours of those who provide informal help to these students need examination. This study examines the helping intentions of undergraduates in Sri Lanka towards their depressed peers and the correlates of their helping intentions.

**Method:**

The undergraduates were presented with a vignette of a hypothetical depressed undergraduate. A total of 4442 undergraduates responded to an open-ended question about how the person in the vignette should be helped if this person was someone they knew well. Their responses were coded in reference to established mental health first aid guidelines. Logistic and linear regression models were used to examine the predictors of their helping intentions.

**Results:**

The undergraduates’ most common helping intentions were to listen/talk and support their peer. Only around a third considered the need for professional help. The overall quality of their helping intentions was poor, but better among those who recognised the problem as depression and those who had less stigmatising attitudes. There was some evidence that certain helping intentions of the undergraduates which were person-oriented or social network-related were better among females, those in higher years of study and among certain non-medical student groups. Intentions to encourage professional help were better among those who recognised the problem, but poorer among those with personal experiences of this problem and among those who perceived this problem to be a *weakness* and not a *sickness*.

**Conclusions:**

Although the undergraduates may attempt to support their distressed peers, they may not show appropriate helping actions and may not encourage the use of professional assistance. Hence, they need to be educated on how best to respond to their distressed peers. Those with higher levels of stigma and inability to recognise the problem may be at greater risk of showing poorer helping responses towards their distressed peers.

**Electronic supplementary material:**

The online version of this article (doi:10.1186/s12888-017-1192-7) contains supplementary material, which is available to authorized users.

## Background

It is concerning that despite the high rates of depression among university students [1, 2] many do not seek the necessary professional assistance [3–9]. Evidence indicates that they show preference to seek help from their informal social network, such as their friends and parents, when dealing with mental health problems [10–15]. Studies also indicate that the social networks of university students could influence their mental health service use [7, 16–18] and play significant roles as gateway providers to treatment [19]. This highlights the need to assess whether these informal help-providers have the appropriate knowledge and skills to respond to distressed university students.

The term ‘mental health first aid’ has been used to describe the help given to an individual who is developing a mental health problem or in a mental health crisis, until appropriate professional treatment is provided or until the crisis situation resolves [20–22]. The social networks of mentally ill persons must possess appropriate mental health first aid knowledge, as they may play important roles with regard to helping these persons to recognise their problem, providing them social support and influencing their help-seeking practices [23].

An assessment of the mental health first aid knowledge and skills of university students is important for several reasons. While these students are frequently faced with friends or family who are affected by mental health problems [24–29], they consider one another as preferred sources of help when dealing with such problems [10–15, 29]. Moreover, they may seek the help of other students when attempting to help distressed peers [30]. However, these students may vary in their confidence about helping their mentally distressed peers [24, 31, 32], and lack confidence in their ability to respond to peers with severe problems [25, 33]. Therefore, the mental health first aid knowledge and skills of university students must be assessed and improved when attempting to ensure that those who are distressed in this population get the appropriate assistance.

This paper focusses on the depression-related mental health first aid responses of undergraduates in Sri Lanka. When depression and depression literacy involving the ability to recognise and respond to the disorder were examined among these undergraduates, the findings indicated that although close to 10% could be depressed [34], they may not seek professional assistance for their depression and instead seek informal assistance, such as help from friends [35]. These findings and the limited mental health services available in the country overall [36–38], highlight the active role that these undergraduates may play as gateway providers and catalysts in ensuring that their distressed peers get the necessary professional assistance. Therefore, an assessment of these undergraduates’ mental health first aid responses is needed.

Many studies examining the mental health-related helping responses of various populations have focussed on their helping intentions [27, 32, 39, 40], with only a few examining their actual helping actions [29, 41]. While the Theory of Planned Behaviour [42] supports the examination of helping intentions as a predictor of helping actions, there is evidence for such a relationship [43, 44].

There have also been a series of Delphi studies undertaken to establish expert consensus guidelines regarding the most appropriate mental health first aid skills for different mental disorders [22, 45–47]. These guidelines have facilitated the development of a Mental Health First Aid Course by Kitchener et al. [48] to educate the public on how to respond to someone developing a mental illness or in a mental health crisis situation. This course, which is Australian-based, is now recognised internationally [49]. Many studies have used these guidelines as a standard to assess the mental health first aid knowledge of various Australian and Japanese populations [20, 29, 40, 41, 50–52]. Kelly et al. [53] developed a scoring system based on these guidelines, enabling the quantification of the quality of helping responses of participants. This methodology has also been used to assess the quality of helping responses of various populations in Australia and Japan [51, 54–56]. While this scoring system was used by Davies et al. [32], to assess the quality of intended helping actions of British university students towards their depressed peers, the overall quality of these students’ helping intentions was found to be poor.

Studies have also identified certain factors which predict the first aid knowledge and related intentions and actions of various populations. For example, intentions and actions relating to providing help to mentally ill persons are found to be generally better among females [31, 32, 39–41, 52, 57], older youth [29, 32, 40, 41] those able to recognise the presence of a mental health problem [39, 52], those with less stigmatising attitudes [32, 52, 57, 58] and those with experiences of mental illness and related help-seeking [25, 32, 40, 52]. An examination of the predictors of the helping responses of the undergraduates would assist in guiding attempts to improve their related actions by identifying groups to be targeted. Therefore, this paper examines both the helping intentions of undergraduates in Sri Lanka towards their depressed peers and the correlates of their helping intentions.

## Method

### Design, participants, setting

This was a cross-sectional study conducted as a part of the previously described depression prevalence and depression literacy survey undertaken among undergraduates in Sri Lanka from June to November 2013. The study was conducted at the University of Colombo. Its methodology has been described previously [35]. The participants were undergraduates in all years of study who attended lectures in five of the six undergraduate faculties of the University of Colombo, namely the Faculties of Arts, Law, Management and Finance, Medicine and Science, as well as the School of Computing, which is an affiliated institute of the university. Data was collected from all undergraduates who attended lectures which were common for each year of study in each of the faculties/schools. However, this was not possible in the case of the Faculty of Arts due to the varied and numerous subject combinations it offered. Therefore, lectures with the largest student groups were approached for data collection.

The strategy of systematically approaching students from all faculties and years of study during the identified lectures was considered to reduce any bias in sampling. This sampling strategy also attempted to obtain a large a sample as possible, to enable an examination of the effects of the examined variables in the sub-groups of the population.

### Measure

The depression literacy survey was administered through a paper-based dual-language questionnaire that was available in two versions, i.e., as English-Sinhala and English-Tamil versions, where participants could choose their preferred version.

#### Variables measured

The questionnaire examined the participants’ depression literacy, relating to their ability to recognise the problem, their treatment beliefs, help-seeking intentions and helping intentions towards a depressed peer; their stigma towards depressed peers in relation to a personal stigma and social distance scale (see Amarasuriya et al. [59]) their exposure to negative life events; and whether they screened positive for Major Depression as per the Patient Health Questionnaire-9 (PHQ-9) [60] (diagnosis given if five or more symptoms in the PHQ-9 were present at least “more than half the days” during the past 2 weeks, with either symptoms of depressed mood or anhedonia. If the symptom of suicidal thoughts was present at all, this contributed to the symptom count for a diagnosis). The questionnaire also included sections examining the participants’ demographic characteristics and previous exposure to depression either through personal experiences or through the experiences of family and friends. Their depression literacy, stigma and prior exposure to the problem were examined using a vignette which described an undergraduate named ‘Z’ who exhibited symptoms which aligned with five of the nine symptoms considered for a diagnosis of Major Depression in the DSM-IV (including depressed mood) over several weeks. The description also indicated impairment in social and occupational functioning. The depression literacy questionnaire, including the depression vignette and details regarding its development, have been published elsewhere [35, 61]. The present paper examines the helping intentions of the undergraduates towards their depressed peers which were elicited using the following open-ended question.

“Imagine that ‘Z’ is someone you have known for a long time and know well. You want to help ‘Z’. What do you think is appropriate to do?”

### Procedure

The questionnaires were distributed to the undergraduates during lectures. Each respective class identified for data collection was approached separately at a time identified by the academic and administrative staff. Participation was voluntary and anonymous. Participants did not receive any remuneration for participation in way of course credits or incentives. Consent to participate was implied when a filled questionnaire was returned. The undergraduates took approximately 20 minutes to complete the questionnaire.

#### Coding helping intentions question

Two strategies were used to code the undergraduates’ responses. The first approach attempted to describe their different types of helping intentions without making any judgements about the quality of their intentions. Accordingly, SDA (first author) created coding categories relevant to all responses varying in meaning. The categories were coded as *yes* or *no*. Multiple categories could be coded. Subsequently, common categories were re-categorised into coding sub-categories. Next, the coding categories / sub-categories were grouped into qualitatively distinctive descriptive categories. The coding scheme utilised by Jorm et al. [52] to descriptively code the helping intentions of the Australian public towards their mentally ill associates was used as a guide.

The second coding strategy examined the quality of the responses provided by those responding in English using a revised version of the Kelly et al. [53] scoring system [43, 55]. These scoring systems are based on the ALGEE action plan that is taught in the aforementioned Mental Health First Aid course [48]. The acronym ALGEE relates to the following helping actions: approach the person (abbreviated as *approach*); assess and assist with any crisis (abbreviated as *assess/assist*); listen non-judgmentally (abbreviated as *listen*); give support and information (abbreviated as *give support*); encourage appropriate professional help (abbreviated as *professional help*); encourage other supports (abbreviated as *other supports*). Responses relevant to these actions were scored for their quality and their degree of detail. Scores for each of the six components of the action plan ranged from 0 to 2: a score of 0 was given if the action was not mentioned or was inappropriate; 1 point was awarded if the helping action was indicated, but in a superficial manner; 2 points were awarded if the quality of the action was indicated or if specific details of the action were provided. A total score indicating the quality and extensiveness of the responses was obtained by summing the points awarded for the six ALGEE components leading to scores ranging from 0 to 12.

SDA coded the responses of those who responded in English using the ALGEE scoring system. In order to establish inter-rater reliability 50 responses of the study sample (2.76% of responses scored using the ALGEE scheme) which were selected using the SPSS random numbers function, were independently scored by SDA, AR (an expert scorer) and AFJ (a developer of the scoring system). The consensus scores of the latter experts were compared with the scores given by SDA. Such a procedure has been used to examine inter-rater reliability of ALGEE scoring in previous studies [43, 53]. Inter-rater reliability between the expert consensus scores and SDA’s scores for the ALGEE components and for the total score were assessed using Pearson’s r and were as follows: *approach*, *r* = 0.86; *listen*, *r* = 0.93; *give support*, *r* = 0.86; *professional help*, *r* = 0.96; *other supports*, *r* = 0.87; Total ALGEE scores, *r* = 0.90; r was not calculated for the component *assess/assist*, as all the selected responses were given a score of 0 for this component by the three scorers. As the inter-rater reliability estimates were high, SDA proceeded to score the other responses of the sample, consulting with the experts whenever necessary.

### Statistical analyses

Percent frequencies and 95% confidence intervals were obtained for the descriptive coding categories. Descriptive statistics were calculated for the ALGEE component scores and the total score.

Binary logistic regression models were used to examine the predictors (IVs) of each of the descriptive coding categories (DVs). Accordingly, the following categorical variables were simultaneously entered into each model, where the variable sub-categories that are italicised were the reference categories for each of the respective variables: gender (*male*, female); age category (*18–20 years*, 21–23 years, 24 years and above); faculty/school of study (*Medicine*, Arts and Education, Law, Management and Finance, Science, School of Computing); year of study (*1st year*, 2nd year, 3rd year, 4th year, 5th year Medicine); ability to recognise the problem in the vignette (*not recognised*, recognised as depression, recognised as a mental health-related problem, where responses were relevant to the label categories “mental illness”, “stress/pressure/mental suffering” or “mental issue”; see Amarasuriya et al. [35] for further details on the response categories); whether the problem in the vignette had been experienced by family or friends (*no*, yes, don’t know); whether the problem was personally experienced (*no*, yes, don’t know); and whether the respondents screened positive for Major Depression as per the PHQ-9 (*no*; yes) [60]. The undergraduates’ stigma in relation to the Personal Stigma and Social Distance scales were also examined as predictors (continuous variables). Amarasuriya et al. [59] found that the former scale consisted of two dimensions of stigma, i.e., the Weak-not-Sick and Dangerous-Undesirable dimensions, and that the latter scale consisted of one dimension, which was labelled as Social Distance. The undergraduates’ stigma scores were entered into the model in relation to scales which were constructed to reflect these three dimensions of stigma (see Amarasuriya et al. [59] for details regarding construction of scales, factor loadings of scale items, reliabilities of scales and their limitations relating to low reliability estimates).

A simultaneous multiple linear regression model was used to examine if the aforementioned variables were predictors of the total ALGEE scores (DV). Accordingly all these variables, except for the stigma scales scores, were dummy-coded using the same reference categories as used in the aforementioned analyses.

All analyses were adjusted for the participants’ religion and residence. The analyses relating to the descriptive coding categories were also adjusted for the response language. Due to the large number of predictors entered into the models, the *p* < .01 level of significance was used to reduce the Type I error rate.

The use of simultaneous regression analysis models allowed for the examination of the associations that each of the examined IVs had with the DVs while adjusting for the effects of other variables. To examine whether these IVs had similar associations with the DVs in the absence of such adjustments, variables found to be significant predictors of the DVs at the *p* < .01 level were examined using univariate regression analysis models.

## Results

### Participant characteristics

From the 4671 undergraduates who participated in the depression literacy survey, 4442 responded to the helping intentions question (229 missing responses). This was approximately 50% of the undergraduate population at the University of Colombo. The responses were provided in English by 40.5%, in Sinhala by 57.0%, in Tamil by 2.0% and in more than one language by the rest. Table 1 presents the demographic and other characteristics of those who responded to the helping intentions question and those whose responses were analysed using the ALGEE scoring system (those who responded in English; *n* = 1811). As seen, approximately 60% of each group indicated that someone in their family or close circle of friends had experienced the problem described in the vignette.

**Table 1:** Demographic and other characteristics of undergraduates who responded to helping intentions question and those whose responses were scored using the ALGEE scoring system

Variables	Undergraduates who responded to helping intentions question		Those whose responses were scored using ALGEE system	
	(*n* = 4442)		(*n* = 1811)	
	n	%	n	%
Gender				
Male	1338	30.1	667	36.8
Female	3100	69.8	1142	63.1
Faculty				
Medicine	566	12.7	522	28.8
Arts and Education^1^	1159	26.1	50	2.8
Law	607	13.7	114	6.3
Management and Finance	980	22.1	460	25.4
Science	631	14.2	411	22.7
School of Computing	498	11.2	254	14.0
Year of study				
1st year	1842	41.5	524	28.9
2nd year	1181	26.6	504	27.8
3rd year	796	17.9	333	18.4
4th year	515	11.6	344	19.0
5th year (Medicine)^2^	108	2.4	106	5.9
Age group				
18–20 years	485	10.9	155	8.6
21–23 years	3189	71.9	1180	65.2
24 years and above	763	17.2	476	26.3
Religion				
Buddhist	3870	87.1	1425	78.7
Hindu	145	3.3	118	6.5
Islamic	146	3.3	79	4.4
Roman Catholic	205	4.6	135	7.5
Other	70	1.6	49	2.7
Residence when going to University				
Home	1662	37.4	973	53.7
Hostel	1338	30.1	335	18.5
Rented place	1126	25.3	390	21.5
Home of friend or relative	262	5.9	81	4.5
Other	50	1.1	30	1.7
Exposure to problem through family/ friends				
No	1684	37.9	632	34.9
Yes	1646	37.1	743	41.0
Don’t know	983	22.1	382	21.1
Personal experience of problem				
No	2395	53.9	968	53.5
Yes	1461	32.9	581	32.1
Don’t know	305	6.9	170	9.4
Screening positive for Major Depression^3^	(*n* = 4107)		(*n* = 1671)	
No	3731	90.8	1555	93.1
Yes	376	9.2	116	6.9

Notes.

^1^ Only 5.6% of the total sample and 2% of those whose responses were scored using the ALGEE scoring system were from the Faculty of Education

^2^ Only the Faculty of Medicine has a 5th year of study

^3^ Only responses which had ≤ 1 missing response on the PHQ-9 were considered valid for this variable

### Helping intentions in relation to descriptive coding categories

The undergraduates’ responses were grouped into the seven categories presented in Table 2. The most common responses were to provide support and to explore the problem. Only 29.6% of undergraduates stated that they would encourage/help their peer to obtain professional help. Also, only low proportions of undergraduates indicated that they would consider their approach to Z or that they would encourage/help Z to either seek informal support or to engage in self-help strategies. The proportion of undergraduates who stated that they would assess whether Z was at any risk of harm was extremely low. Although some indicated that they would “keep an eye on Z” (approximately 1%), these responses were not coded for assessing risk for harm, unlike in the Jorm et al. [52] study, as it was not clear whether these responses were relevant to this specific action or merely the respondent’s attempt to provide more attention to Z than earlier. The correlations between the identified descriptive categories are presented in Additional file 1.

**Table 2:** Descriptive categorisation of helping intentions

Descriptive category	Coding sub-categories	% of responses (95% CI) (*n* = 4442)
Consider approach to person	set up the context; become close or trustworthy to the person; engage the person; indicate concern and readiness to talk	6.9 (6.1–7.6)
Assess risk of harm	assess for risk of harm to oneself or others	0.1 (0–0.2)
Explore problem	listen and/or talk about the problem; attempt to understand problem	52.1 (50.6–53.5)
Provide support	provide emotional comfort and encouragement; associate with the person closely; provide advice and opinions; brain storm; give information; help the person (e.g., with daily activities, needs); do something to help	64.9 (63.5–66.3)
Encourage/help to seek professional help	help from: psychiatrist/related help; psychologist; counsellor/counselling; doctor/ medical assistance/medicine; student counsellor; university medical officer; mental health professional at university psychiatry unit; unspecified mental health professional; professional; treatment	29.6 (28.3–31.0)
Encourage/help to seek informal help	help from: parents/family; friends; elders; someone close to the person; university personnel; help to socialise/interact with friends/ others	9.8 (9.0–10.7)
Encourage/help in self-help strategies	enjoyable/relaxing/extra-curricular activities; take a break; religious activities; meditation; self-help books and movies; distraction and or distancing from problem	10.6 (9.7–11.5)

Note.

^1^ The percentages add up to more than 100% as responses could be coded across multiple categories

Counsellors/counselling was the type of professional help most nominated (10.9%), followed by psychiatrists/related help (8.4%) and doctors/getting medical assistance (6.9%). A relatively large proportion of responses relevant to the descriptive category of giving support were about providing advice or one’s opinions to Z (28.4%). While many responses only indicated that advice would be given, there were others that included details about the advice. The latter responses included advice about how Z should respond to the problem, advice about life that sometimes had a philosophical stance, advice to encourage or to think positively, advice to not think too much / worry about the problem, advice attempting to normalise/minimise the problem, advice based on personal experiences or experiences of others, advice about studies, advice to relax or take a break, religious-oriented advice, advice on physical and psychological wellbeing and an explanation of the behaviour or situation to Z. While these responses were varied, as seen in the following examples, they also varied in quality with regard to their helpfulness or potential harmfulness.

Example 1: “Ask from her about the problem she has and try to make her mind, that everyone can face such problems during life.” (response 433)

Example 2: “I will listen to her problem and convince her that it is a small problem compared to some problems that some people have.” (response 4643)

Both Examples 1 and 2 portray attempts to normalise the problem. However, the response in Example 2 may also reflect minimisation of the problem and disregard for the individual’s personal experiences relating to it and hence, be potentially harmful.

### Correlates of descriptive coding categories

Table 3 presents the adjusted odds ratios (ORs) for predictors which were found to be significant at *p* < .01. Both the adjusted and unadjusted ORs for these predictors show a similar trend. Correlates for the coding category relating to assessment of risk of harm were not examined due to the low frequency of these responses.

**Table 3:** Correlates of helping intentions as per descriptive coding categories using binary logistic regression

Predictor variables	Approach person	Explore problem	Provide support	Encourage/help to get professional help	Encourage/help to get informal help	Encourage/help to engage in self-help
	Adjusted and unadjusted^+^ OR (99% CI)	Adjusted and unadjusted^+^ OR (99% CI)	Adjusted and unadjusted^+^ OR (99% CI)	Adjusted and unadjusted^+^ OR (99% CI)	Adjusted and unadjusted^+^ OR (99% CI)	Adjusted and unadjusted^+^ OR (99% CI)
Gender (reference group: Male)						
Female		1.43^***^ (1.17, 1.75) *1.44* ^***^ *(1.21, 1.70)*			1.46^**^ (1.02, 2.09) *1.41* ^**^ *(1.04, 1.90)*	
Faculty (reference group: Medicine)						
Management and Finance			1.52^**^ (1.02, 2.24) *2.65* ^***^ *(2.00, 3.52)*			
Year (reference group: 1st year)						
2nd Year	0.58^**^ (0.36, 0.93) *0.62* ^***^ *(0.42, 0.91)*				0.59^**^ (0.39, 0.90) *0.63* ^***^ *(0.45, 0.88)*	
3rd Year			0.74^**^ (0.55, 0.99) *0.68* ^***^ *(0.55, 0.86)*			
5th Year (Medicine)			0.32^***^ (0.14, 0.73) *0.20* ^***^ *(0.11, 0.34)*	2.39^**^ (1.06, 5.38) *4.47* ^***^ *(2.63, 7.62)*		
Exposure to problem through personal experience (reference group: response: no)						
Response: Yes			1.26^**^ (1.00, 1.58) *1.40* ^***^ *(1.17, 1.68)*	0.73^**^ (0.57, 0.93) *0.64* ^***^ *(0.52, 0.77)*		1.47^**^ (1.05, 2.04) *1.46* ^***^ *(1.12, 1.92)*
Recognition of problem in case vignette (reference group: not recognised)						
Recognised as depression			0.49^***^ (0.36, 0.68) *0.29* ^***^ *(0.22, 0.37)*	3.26^***^ (2.32, 4.59) *4.29* ^***^ *(3.26, 5.63)*		
Recognised using other mental health-related label			0.54^***^ (0.43, 0.68) *0.59* ^***^ *(0.48, 0.72)*	2.22^***^ (1.72, 2.86) *2.50* ^***^ *(1.99, 3.14)*		
Stigma scale scores						
Weak-not-Sick Scale			1.11^***^ (1.07, 1.16) *1.14* ^***^ *(1.10, 1.18)*	0.88^***^ (0.85, 0.92) *0.86* ^***^ *(0.83, 0.89)*	0.92^**^ (0.86, 0.98) *0.94* ^**^ *(0.89, 1.00)*	
Dangerous-Undesirable Scale			0.93^**^ (0.88, 0.98) *0.91* ^***^ *(0.87, 0.95)*			
Social Distance Scale						0.94^**^ (0.89, 1.00) *0.94* ^**^ *(0.89, 0.99)*
Nagelkerke *R* ^2^ (when all variables are entered simultaneously into model)	0.04	0.03	0.12	0.11	0.05	0.04

Notes.

^**^ *p* < .01,^***^ *p* < 0.001

^+^ Unadjusted ORs and related 99% CIs have been italicised

*n* = 3740 for analyses where adjusted ORs were obtained; *n* = 4161 – 4442 for analyses where unadjusted ORs were obtained

- Female undergraduates had higher odds of intending to explore the problem and of encouraging/helping Z to obtain informal help.
- Faculty of study was only a predictor with regard to giving support, where odds of intending to give support were higher among Management and Finance undergraduates as compared to Medical undergraduates.
- In comparison to 1st year undergraduates, while those in the 2nd year had lower odds of indicating their approach to Z and intending to encourage/help Z to get informal help, those in the 3rd year had lower odds of intending to provide support. Compared to the first-years, 5th year Medical undergraduates had lower odds of indicating that they would provide support to Z but higher odds of intending to encourage/help Z to get professional help.
- Those who had personally experienced the problem had higher odds of intending to provide support and of encouraging/helping Z to engage in self-help strategies, but lower odds of indicating that they would encourage/help Z to get professional help.
- Those who recognised the problem as depression or used other mental health-related labels for the problem had lower odds of indicating that they would provide support, but higher odds of indicating that they would encourage/help Z to get professional help.
- Those who had higher scores on the Weak-not-Sick scale had higher odds of indicating provision of support, but lower odds of intending to encourage/help Z to get both professional and informal help. Those who had higher scores on the Dangerous-Undesirable scale had lower odds of intending to provide Z support. Those who had higher scores on the Social Distance scale had lower odds of intending to encourage/help Z to engage in self-help activities.

### ALGEE scores of those who responded in English

A total of 1811 responses were analysed. Scores ranged from 0 to 7 out of a maximum of 12. Respondents obtained a mean score of 2.13 (SD = 1.09; Median = 2.00). The means and SDs of the scores for the different ALGEE components were as follows: *approach* (Mean = 0.06; SD = 0.25); *assess and assist* (Mean = 0; SD = 0.05); *listen* (Mean = 0.58; SD = 0.63); *give support* (Mean = 0.66; SD = 0.57); *professional help* (Mean = 0.64; SD = 0.91); *other supports* (Mean = 0.19; SD = 0.41). The correlations between these different components are provided in Additional file 2.

The response pattern of this sub-set of undergraduates was similar to that observed among the overall sample. The ALGEE components nominated the most were *give support* (61.2%) and *listen* (50.4%). Only around one third of undergraduates obtained points for encouraging *professional help* (33.4%). Less than one fifth obtained points for encouraging *other supports* (18.3%). The percentage of those who considered their *approach* was much lower (5.6%). A negligible percentage obtained points for *assess/assist* (0.2%).

When considering the quality of the responses as indicated by the mean scores obtained for the ALGEE components, response quality for the component *professional help* (0.64) was slightly lower than that for the component *give support* (0.66), but higher than that for the component *listen* (0.58). Furthermore, the highest occurrences of a maximum score of 2 points were seen for the component *professional help* (30.1%). The percentage of responses awarded 2 points for the *give support* and *listen* components were comparatively lower (5.0 and 7.9% respectively).

### Unhelpful responses

A dichotomous coding category labelled “unhelpful” [57] was created to code the potentially harmful responses (coded as *yes/no*). This contained a total of 30 responses (1.66% of responses scored using ALGEE scheme), including unhelpful advice or attempts to minimise the problem, coercion to seek treatment and encouragement of alcohol/substance use.

### Correlates of total ALGEE scores

Table 4 presents the adjusted standardised regression coefficients relevant to the variables which were found to be significant predictors of the total ALGEE scores at *p* < .01. Both the adjusted and unadjusted regression coefficients show a similar trend. Those who recognised the problem as depression had higher ALGEE scores. Those in the 3rd year (compared to first-years) and those who obtained higher scores on the Weak-not-Sick and Social Distance Scales had lower ALGEE scores.

**Table 4:** Correlates of ALGEE scores using linear regression

Predictor variable	Adjusted and unadjusted ^+^ standardised regression coefficient (99% CI)	
Year (reference group: 1st year)		
3rd Year	−0.11^**^ −*0.07* ^**^	(−0.20, −0.03) *(−0.13, −0.01)*
Recognition of problem in case vignette (reference group: not recognised)		
*not recognised*		
Recognised as “depression”	0.13^***^ *0.10* ^***^	(0.05, 0.22) *(0.04, 0.16)*
Stigma Scale Scores		
Weak-not-Sick Scale	−0.12^***^ −*0.12* ^***^	(−0.18, −0.05) *(−0.18, −0.06)*
Social Distance Scale	−0.10^***^ −*0.09* ^***^	(−0.17, −0.03) *(−0.16, −0.03)*
Adjusted R^2^ (when all variables are entered simultaneously into model)	0.06	

Notes.

^**^ *p* < .01, ^***^ *p* < 0.001

^+^ Unadjusted regression coefficients and related 99% CIs have been italicised

*n* = 1543 for analyses where adjusted regression coefficients were obtained; *n* = 1786–1811 for analyses where unadjusted regression coefficients were obtained

## Discussion

This study examined the helping intentions of undergraduates in Sri Lanka towards a depressed peer (described in a vignette) and the correlates of their helping intentions. The finding that approximately 60% of the undergraduates had encountered the problem among their family or friends highlights the importance of this examination. The undergraduates’ most common helping intentions were to provide support and to listen and/or talk to their depressed peer. Only approximately 30% considered recommending professional help. The quality of the helping intentions of those who responded in English was also poor. The undergraduates’ ability to recognise the problem and their stigma towards their peers predicted their helping intentions with regard to both the coding strategies that were used. Following is a more detailed discussion of the findings.

### Helping intentions of undergraduates

While these undergraduates’ helping intentions seem to be poor as per the ALGEE scoring system, their mean quality score (M = 2.13) is also somewhat lower than the score obtained by British university students (M = 2.83) in the Davies et al. study [32]. This indicates the need to improve their knowledge about assisting their mentally distressed peers, given the rate of depression among this population [34] and their endorsement of their peers as help-providers when dealing with depression [35]. However, their mean ALGEE score is similar to that obtained by Australian adults [55] and higher than that obtained by adolescents in Australia [43] and Japan [51]. This highlights a trend of poor mental health first aid knowledge across various population groups.

As in other studies among university students, this study also indicates that the most common helping responses of undergraduates towards their distressed associates are to listen and/or talk and support the affected person [29, 30, 32]. However, there was a considerable proportion who did not indicate such actions. Furthermore, when considering the subset of responses scored using the ALGEE scoring system, only low proportions of undergraduates indicated the quality of their interaction and communications with their peer (7.9%) or considered diverse ways of providing support (5.0%). Therefore, it is important to ensure that these undergraduates are aware of how best to interact with distressed peers.

Furthermore, the undergraduates’ intentions to recommend professional help seem to be low when considering the findings of similar studies, such as that by Davies et al. [32] among British university students and those among Australian adults [52, 55], which have found that between 50 and 60% of participants consider the need for professional help for a depressed associate. Hence, these undergraduates need to be educated about the importance of referring their distressed peers to professional treatment to thereby empower them as gateway providers to such treatment [19]. It is interesting to note, however, that although university students in contexts such as Australia consider the help of specialists, such as psychiatrists, to a much lesser extent than the help of professionals such as General Practitioners (GP) for depression [12, 29], psychiatrists were among the professional categories that were most commonly nominated by the present study sample. These differences in findings may reflect differences in the healthcare systems of the two study sites. The study findings align with what is seen in China where, in the absence of GP-based systems, the population prefers to consult specialists via the hospitals [11].

A noteworthy finding was that among those who indicated that they would give support to their depressed peer, a relatively large proportion stated that they would give advice. It would be interesting to examine whether cultural factors, such as the collectivist culture in Sri Lanka, influence the undergraduates’ receptivity to such advice [62, 63]. While it could be assumed that the intention of those who indicated that they would provide advice was to assist their peer, it was noted that some of their advice could be potentially harmful. As advice-giving may be a common form of support in this population, it is important to provide guidance to these undergraduates about the considerations to make when giving such advice [64, 65].

Actions the undergraduates rarely considered were assessment of their peers’ risk of being at harm and provision of assistance to deal with any crises. Similar results have been observed in other similar studies among various population groups [32, 43, 55]. However, the vignette used in the present study did not indicate that the undergraduate who was described was at any risk of harm or in any crisis; this may explain the findings. Nevertheless, these undergraduates need to be aware of the necessity of being vigilant about whether their distressed peers are at risk of harm and especially at risk of self-harm as suicide rates have been high in Sri Lanka, although they are now on the decline [66]. Given the findings that these undergraduates are likely to seek assistance from their peers for their problems [35], it is also important to ensure that this population is able to respond appropriately to those at risk of self-harming behaviours. The aforementioned issue of harmful advice may need particular attention, as this could be detrimental to help-seeking among such individuals.

### Correlates of helping intentions

The undergraduates’ ability to recognise the problem as depression was associated with them showing better helping intentions with regard to both coding systems. When considering the results relevant to the descriptive coding categories, as seen in previous research [52], those able to recognise the problem were more likely to consider the need for professional help. However, these individuals were less likely to indicate that they would provide support to their depressed peer. Given the methodology of open-ended questions, it is not possible to establish whether problem-recognition is associated with the undergraduates being less inclined to support their depressed peers or whether it is associated with them being more likely to indicate the need for professional help as compared to provision of support. There is some evidence seen for the latter possibility in relation to the weak negative correlations found between responses relating to encouraging professional help and providing support to Z (descriptive categories: *r* = −0.38. *p* < .001; ALGEE scoring system: *r* = −0.18, *p* < .001), which might indicate that identifying the need for professional help is associated with less inclination to consider providing personal support. A similar pattern of being more likely to recommend professional help but less likely to provide support was seen among 5th year medical undergraduates, who are in their final year of undergraduate medical training and potentially more likely to recognise the need for professional assistance for the problem.

The study findings concur with previous research which indicates that stigma predicts the helping intentions of participants [32, 52, 57, 58]. It is noteworthy that the findings from the entire undergraduate sample indicate that those who perceive the problem as a “weakness” and not a “sickness” are more likely to give support but less likely to recommend help from both professional and informal sources. This highlights the importance of the undergraduates acknowledging the problem as a *real illness*, as this seems to be associated with their understanding of the need to direct their distressed peers to obtain the necessary help.

Although previous studies using the ALGEE scoring system have found the quality of helping responses of females to be better [32, 57], the present study found no such gender differences. This indicates that both males and females in this undergraduate population need equal attention when educating them about assisting their mentally distressed peers. However, females were more likely to express their intentions to engage in certain actions, such as exploring the problem of their depressed peer and encouraging/helping them to get informal help. This greater propensity among female undergraduates to engage in person-centred and social-network-related approaches may be a by-product of gender-related differences, where females show greater confidence to support a friend with mental health problems, have higher levels of empathy and greater skills to provide emotional support [24, 67, 68].

A surprising finding was that medical undergraduates did not exhibit better helping intentions than their non-medical peers, especially with regard to their intentions to recommend professional help and in relation to their total ALGEE scores. Davies et al. [32] also found that although university students in clinically-relevant degrees had better overall ALGEE scores, they did not differ from the others in their intentions to recommend professional help to a depressed peer. Such findings highlight the need to examine whether medical undergraduates are reluctant to recommend professional help to their distressed peers and whether this may be associated with their perception that help-seeking for mental health problems would negatively reflect on their competence to practice [69–71].

Those in higher years of study may also be reluctant to provide support to their depressed peers. As the findings provide only some evidence that those in higher years of study show poorer helping intentions, further examination is needed of the changes in the helping intentions of these undergraduates as they progress through their undergraduate studies.

Another area to explore would be the response pattern among undergraduates reporting personal experiences of the problem. Even though they seem more likely to recommend self-help strategies and to provide support, they seem less likely to encourage/help distressed peers to get professional help. These findings could be due to those with a shared history of the problem feeling better able to assist and empathise with those experiencing the problem and being motivated to provide help on their own [27]. However, these findings could also be due to the phenomenology of depression of those with personal experiences of the problem being associated with pessimistic expectations about treatments for depression. Further examination of the reasons for these findings is needed.

The undergraduates could benefit from programmes such as the Mental Health First Aid Course developed by Kitchener et al. [48] to improve their knowledge about how to assist someone with a mental illness. Studies show that this course results in better recognition of disorders, greater alignment with professionals’ beliefs about treatments, decreased social distance from the mentally ill, increases in confidence to provide help to mentally ill individuals and an increase in actual help provided [72–74]. Improvements in helping intentions and mental health literacy and decreases in stigma have also been observed when this course was delivered among nursing and medical students in Australia [75].

The limitations of the study must also be considered. The regression models only accounted for a small percentage of variance in helping intentions, indicating that there are other variables which may need to be considered when attempting to predict the helping responses of this population. Because of the number of significance tests carried out, there may have been Type I errors. However, the use of the more conservative alpha level of .01 means that less than 1 of the tests reported in Tables 3 and 4 would be expected to be significant if the null hypotheses were all true, whereas 20 were actually significant in the case of Table 3 and four were significant in the case of Table 4. However, the small effect sizes of some of the examined relationships indicated by the odds ratios [76], standardised regression coefficients and R^2^ estimates [77] indicate that the practical significance of these associations may be small. Furthermore, the vignette of depression used as the stimulus in the study questionnaire may have been unable to capture the complex interplay of factors that influence the helping intentions of the undergraduates in real life. As this study only examined helping intentions, the findings need to be interpreted with caution, given the evidence that certain intentions, such as encouragement of professional help, may not translate into actual behaviours [43]. Nevertheless, as described by Jorm et al. [52], the helping intentions reported by the participants could be considered to place an upper limit on their actual responses where if they fail to state their intentions relating to a particular action, it is unlikely that they would exhibit this in real-life. However, it must also be considered whether some of the helping actions included in the ALGEE scoring system, such as considering one’s approach to the person, may have been regarded as implicit actions when engaging with the person and not mentioned by the undergraduates. This may explain the relatively low ALGEE scores obtained by the participants and those in previous studies. The analyses relating to the ALGEE scoring could have been subject to sampling bias as these focused only on responses provided in English. Furthermore, there may have been variables, such as previous education in mental health first aid, which were not controlled for in the analyses. Future work must also consider the need to adapt the scoring system to assess certain types of responses that it does not currently assess, e.g., advice responses of varying quality and unhelpful/harmful responses. Although this study was only conducted in one University in Sri Lanka, the large sample size, including undergraduates from diverse disciplines and all years of study, and its reflection of the demographic composition of the undergraduate population in Sri Lanka [78], indicate that the findings provide a useful estimate of the depression-related helping intentions undergraduates in Sri Lanka.

## Conclusions

The findings indicate that the undergraduates would attempt to assist distressed peers by listening and/or talking to them and by providing support. It is however concerning that most undergraduates may not find it necessary to encourage professional help among their distressed peers and may be unaware that their peers could be at risk of harm. Furthermore, they may only consider some of the recommended actions identified in established mental health first aid guidelines and the overall quality of their responses may be poor. Those who recognise the problem and those with lower stigma might show better helping intentions. Gender, year and faculty of study and personal experience of the problem might also be associated with some of their helping intentions.

## Additional files

Additional file 1: Correlations between descriptive coding categories. (XLSX 9 kb)
Additional file 2: Correlation between ALGEE components. (XLSX 10 kb)

## Abbreviations

DV: Dependent variable
GP: General practitioner
IV: Independent variable
OR: Odds ratio
PHQ-9: Patient Health Questionnaire-9
